# 1146. Real-World Experience Highlighting Tocilizumab Use in the Treatment of COVID-19

**DOI:** 10.1093/ofid/ofac492.984

**Published:** 2022-12-15

**Authors:** Christina Maguire, Adrienne Terico, Hinal Patel, George L Anesi, Kathleen Degnan, Lauren Dutcher, Keith W Hamilton, Nuala J Meyer, Naasha J Talati, Steve Saw

**Affiliations:** Penn Presbyterian Medical Center, Philadelphia, Pennsylvania; Pennsylvania Hospital, Philadelphia, Pennsylvania; Penn Medicine Princeton Medical Center, Princeton, New Jersey; Division of Pulmonary, Allergy, and Critical Care, Department of Medicine, University of Pennsylvania Perelman School of Medicine, Philadelphia, Pennsylvania; University of Pennsylvania Perelman School of Medicine, Philadelphia, Pennsylvania; University of Pennsylvania Perelman School of Medicine, Philadelphia, Pennsylvania; University of Pennsylvania Perelman School of Medicine, Philadelphia, Pennsylvania; Division of Pulmonary, Allergy, and Critical Care, Department of Medicine, University of Pennsylvania Perelman School of Medicine, Philadelphia, Pennsylvania; Penn Presbyterian Medical Center, Philadelphia, Pennsylvania; Hospital of the University of Pennsylvania, Philadelphia, Pennsylvania

## Abstract

**Background:**

Tocilizumab (TCZ) was approved by the Food and Drug Administration under emergency use authorization for treatment of COVID-19 in patients requiring supplemental oxygen, non-invasive or invasive mechanical ventilation, or extracorporeal membrane oxygenation. Despite multiple clinical trials, there remain unanswered questions surrounding TCZ use.

**Methods:**

This multi-hospital retrospective cohort study included patients who received TCZ for COVID-19 between January 29^th^, 2021 and June 30^th^, 2021 at five University of Pennsylvania Health System (UPHS) hospitals. Patients were eligible for TCZ per UPHS criteria if they scored ≥ 5 on the World Health Organization (WHO) ordinal scale for ≤ 24 hours and experienced < 14 days of acute COVID-19 symptoms. Descriptive statistics were performed to characterize usage within the health system.

**Results:**

This study evaluated 134 patients who received TCZ for the treatment of COVID-19. TCZ was ordered a median of 22 hours (interquartile range [IQR], 13.2 – 41.5) after hospital admission. A majority of patients (76.1%) were admitted to the intensive care unit and a small portion (12.7%) had a WHO ordinal scale that was >5 at time of TCZ order entry. All patients received concomitant dexamethasone therapy at a total prednisone equivalent of 400 mg (IQR, 335.6 – 480). Overall 33.6% of patients experienced an adverse event (ADE) within 30 days of TCZ administration (Table 1). Most common ADEs included bacterial infection (29.9%), hepatitis (6.7%), and fungal infection (3%); other etiologies of ADEs were not accounted for. All-cause mortality (Table 2) at day 30 occurred in 20.9% of patients and median time from TCZ administration to mortality was 12.5 days (IQR 5 – 18.3). Ninety-six patients in the cohort (71.6%) were discharged by day 30. Of the subgroup discharged by day 30, the majority (70.8%) were discharged to home.

**Figure:**
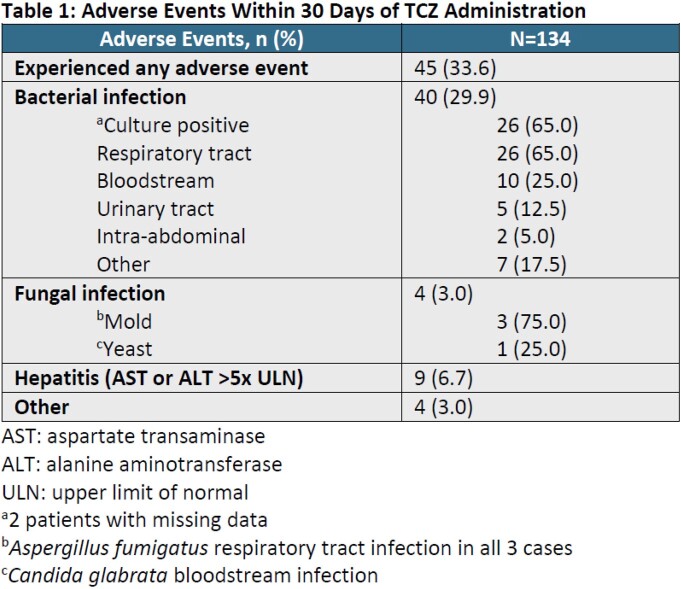

**Figure:**
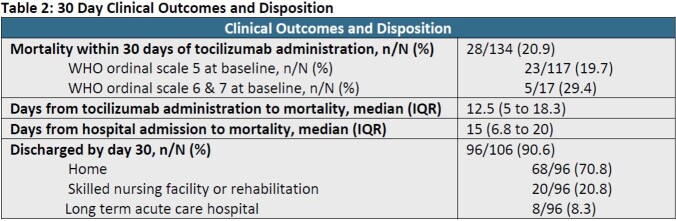

**Conclusion:**

Patients who received TCZ for severe COVID-19 experienced 20.9% mortality; mortality was higher among those with higher ordinal scale at the time of TCZ dosing. A large portion of patients (70.8%) were discharged to home within 30 days. One third of patients experienced an adverse event, primarily bacterial or fungal infection. Our experience may be useful in counseling patients about anticipated effects of TCZ.

**Disclosures:**

**George L Anesi, MD, MSCE**, AHRQ, NHLBI, UpToDate: Advisor/Consultant|AHRQ, NHLBI, UpToDate: Grant/Research Support **Kathleen Degnan, MD**, Gilead: Grant/Research Support.

